# Monocyte subtype expression patterns in septic patients with diabetes are distinct from patterns observed in obese patients

**DOI:** 10.3389/fmed.2022.1026298

**Published:** 2023-01-05

**Authors:** Dan Ning, Kunal Garg, Benjamin Mayer, Benedikt Schick, Hendrik Bracht, Eberhard Barth, Manfred Weiss, Chen Li, Julian Schneider, E. Marion Schneider

**Affiliations:** ^1^Clinic for Anaesthesiology and Intensive Care Medicine, Ulm University Hospital, Ulm, Germany; ^2^Faculty of Medicine, Institute of Epidemiology and Medical Biometry, Ulm University, Ulm, Germany

**Keywords:** sepsis, type 2 diabetes, obesity, monocyte subsets, CD14, CD16, CD163

## Abstract

**Background:**

Sepsis causes a high rate of mortality and long-term morbidity, associated with an imbalance of innate immunity against infections and inflammation. Obesity and diabetes increase the risk for disease severity. Monocyte dysfunction plays a major role and justify further investigations.

**Objective:**

To investigate the distribution and inflammatory phenotypes in circulating monocyte subsets in patients manifesting with sepsis including septic shock with and without obesity and diabetes.

**Methods:**

A total of 235 blood samples were tested from critically ill adult patients registered at the intensive care unit (ICU). The cohorts were divided into non-diabetic groups with or without obesity and diabetic groups with or without obesity, suffering from sepsis or septic shock. We determined frequencies of total monocytes and of monocyte subsets in the circulation and density expression levels of functional markers, including CD14, CD16, HLA-DR, CD33, CD163, CD206, and arginase-1 by flow cytometric analysis.

**Results:**

When progressing to septic shock in non-diabetic and diabetic patients, the percentages of total monocytes among the leukocyte population and of CD33+ and CD14+ monocytes among the monocyte population were consistently down-regulated compared to non-sepsis in non-diabetic and diabetic patients, respectively. Non-diabetic sepsis patients further presented with decreased CD33 and up-regulated CD163 expression density, which was absent in diabetic patients. We subsequently addressed obesity-related changes of monocytes in non-diabetic and diabetic septic patients. Obese septic patients with diabetes were unique in displaying increased monocytic CD16 and CD163 expression. However, obese septic patients without diabetes solely presented with lower amounts of non-classical monocytes. Body mass index (BMI) dependent changes were restricted to diabetic septic patients, with a significantly higher diminution of the classical monocyte subset and concomitantly increased CD16 expression densities.

**Conclusion:**

Distribution and phenotypes of monocyte subsets were differentially modulated in critically ill patients with and without metabolic disease when progressing to sepsis or septic shock. Only diabetic septic patients displayed decline of classical monocytes and increase of CD16 expression densities. Therefore, diabetes but not obesity appears to promote the inflammatory phenotype of circulating monocytes in critically ill patients.

## Introduction

Sepsis, obesity, and diabetes mellitus pose a serious risk to global public health. Sepsis is a life-threatening disease that involves abnormal inflammation and organ dysfunction triggered by an infection (1). The risk for sepsis severity and a higher mortality is further complicated by obesity and diabetes mellitus (2). These metabolic disorders are invariably associated with an impaired monocyte function and are susceptive to infection (3, 4). Clinical evidence demonstrated high morbidity and mortality in obese and diabetic individuals due to sepsis (5, 6). Therefore, it is important to elucidate the role of immune cells like monocytes that promote immunological imbalance in individuals with sepsis, obesity, and/or diabetes mellitus. Studies into sepsis patients and animal sepsis models indicated that a subpopulation of blood monocytes undergo reprogramming that changes their counts and phenotypes (7–9). However, pathological effects of obesity and diabetes mellitus with sepsis on circulating monocytes in humans remain a matter of research interest. Further research is warranted to reveal biomarkers pivotal for the prognosis or diagnosis of sepsis in obese and diabetic patients.

Human peripheral blood monocytes arise from the bone marrow and represent approximately 5–10% of circulating leukocytes. They play a crucial role in phagocyting microbes, clearing apoptotic and necrotic cells, and activating the adaptive immune system (10). For example, monocytes can differentiate into mature macrophages or dendritic cells and utilize the major histocompatibility complex class II molecules to activate CD4^+^ T lymphocytes (6). Monocytes display high plasticity and profound heterogeneity in their typical phenotypes and functions (9, 11–13). Circulating monocytes in humans are grouped into three distinct subsets based on the surface expression of CD14 [a co-receptor for lipopolysaccharides (LPS)] and CD16 (FcγRIII). Firstly, classical monocytes (CD14^+^CD16^–^) account for approximately 90% of circulating monocytes and are crucial for phagocytosis at any infection site (9, 12). The second and third subsets include intermediate (CD14^+^CD16^+^) and non-classical (CD14^low^CD16^+^) monocytes, respectively, that co-express CD16^+^ and exhibit pro-inflammatory characteristics.

Monocytes with CD16^+^ are generally elevated in a variety of diseases, including obesity, diabetes mellitus, cardiovascular disease, trauma, sepsis, autoimmunity, and psychosis (9, 14). The precise onset of sepsis in obese patients with diabetes (5) is challenging, since we lack an understanding of the pathogenic mechanisms driving sepsis (15). To bridge this knowledge gap, we aim to evaluate two points, (1) modulation of the monocyte/macrophage subsets and their function in obese and type 2 diabetes individuals experiencing sepsis, and (2) clinical correlation between the altered monocyte/macrophage cells in a chronic metabolic setting and acute inflammation. The present study elucidated monocyte subset alterations in sepsis patients that were either obese, diabetic (i.e., type 2 diabetes mellitus), or both.

## Materials and methods

### Study design

Between September 2018 and February 2020, a cohort of 235 adult patients were enrolled from the intensive care unit (ICU) of Ulm University Hospital. The cohort included 81 patients with type 2 diabetes mellitus and 47 patients with obesity. Our study protocol (479/20) was approved by the ethics committee at Ulm University. The specimen de-identification process included recording of clinical data [i.e., age, diagnosis, body mass index (BMI), and pre-morbidities] and flow cytometric analysis with whole blood. We did not include samples from pregnant women or individuals with a documented history of allergic disease, autoimmune disease, hepatitis B, hepatitis C, or treatment with glucocorticoids. Diagnosis for diabetes mellitus followed either patient history or American Diabetes Association criteria (16). Diagnosis for sepsis and septic shock complied with The Third International Consensus Definitions for Sepsis and Septic Shock (17). Based on the BMI (kg/m^2^) definition by the World Health Organization (18), patients were identified as underweight (BMI < 18.5 kg/m^2^), lean (BMI 18.5–24.9 kg/m^2^), overweight (BMI ≥ 25 and ≤ 29.9 kg/m^2^), or obese (BMI ≥ 30 kg/m^2^). Specimens were broadly categorized as non-diabetic or diabetic with each group containing non-obese (BMI < 30 kg/m^2^) and obese (BMI ≥ 30 kg/m^2^) cases (Supplementary Table 1). Furthermore, all non-diabetic and diabetic patient groups were sub-divided into non-sepsis, sepsis, and septic shock considering septic severity (Supplementary Table 1). However, due to the small sample size, the obese non-diabetic group was not divided into sepsis and septic shock subgroups, rather presented as a combined “septic” group, as well as the obese and non-obese diabetic groups. The non-sepsis group tested in the current study was selected on the basis of their submission to ICU treatment. Their stay on ICU ranged between 2 and 73 days (mean: 24 days). Mortality rate was 19.6%. ICU patients with no signs of sepsis were admitted with subarachnoidal, intracranial bleeding and ischemia (*n* = 18); surgical or polytrauma (*n* = 31), Leriche syndrome (*n* = 5), unclear pneumothorax (*n* = 3), Fournier Gangrene (*n* = 3), esophagal, aortic, thoracal resection (*n* = 17), surgery due to malignancies (*n* = 18),myocardial infarction, stent implantation (*n* = 8), hemorrhage and bleeding (*n* = 5), pneumothorax, pleuraempyem, pulmonary embolism (*n* = 7), kidney rupture (*n* = 1), pancreatitis (*n* = 1); in residual cases the origin for inflammation and critical illness decompensation could not be specified. Leukocyte counts in the non-septic group of patients ranged between 3.1 and 32.4 G/l (mean: 11.5 G/l) leukocytes/μl. CRP ranged between 0.3 and 187 mg/l (mean: 62 mg/l).

Stay on ICU of the 49 patients with sepsis ranged between 7 and 78 days (mean: 40 days). Mortality rate was 18.4%. A total of *n* = 14 patients suffered from lung disease; 8 patients developed sepsis from abdominal infections, *n* = 7 patients due to urogenital infections, 2 became septic upon cardiac surgery, 2 were related to malignancies and surgery, and 6 had other skin and wound infections which progressed to sepsis. Two patients became septic after brain surgery. The cause of sepsis in residual patients remained undefined but may be related to immune insufficiency, overweight and diabetes. Leukocyte counts ranged between 4.1 and 24 G/l (mean: 12.1 G/l). CRP ranged between 35.5 to 345 mg/l (mean: 133.4 mg/l).

Stay on ICU of the 26 patients with septic shock ranged between 2 and 122 days (mean: 50 days, range: 2–122 days). Nine patients died (mortality rate: 34. 6%). A total of *n* = 10 patients suffered from pneumonia, *n* = 11 patients developed septic shock with urinary tract infections and from severe infectious complications due to wounds, catheter and skin; *n* = 6 due to abdominal surgery. Two patients manifested septic shock after major polytrauma. Individual septic shock cases occured patients with brain abscess (*n* = 1), malignancy (*n* = 1), myositis (*n* = 1), pancreatitis (*n* = 2). The patient with longest stay on ICU (122 days) was admitted with septic embolism of the spleen, then mesenterial ischemia and massive staphylococci in the mesenterial artery. He had survived a renal Hanta virus infection 1 year before his stay on ICU. Leukocyte counts in the septic shock group ranged between 1.9 and 44.0 G/l (mean: 13.1 G/l) leukocytes/μl. CRP ranged between 46.7 and 472.0 mg/l (mean: 190.9 mg/l).

### Flow cytometry analysis to detect an inflammatory phenotype

Phenotype analysis was performed promptly after receiving fresh whole blood samples in ethylenediaminetetraacetic acid anti-coagulation tubes. Briefly, 40–100 μl blood was mixed with 5 μl of each surface protein labeling antibody for 30 min at 4°C in the dark. The antibodies used for labeling included Leucogate™ (CD45/CD14, BD Biosciences, Franklin Lakes, NJ, USA), fluorescein isothiocyanate (FITC)-conjugated anti-CD14 (Diaclone, Besancon, France), anti-CD163 (BD Biosciences), anti-arginase-1 (R&D Systems, Minneapolis, MN, USA), phycoerythrin-conjugated anti-CD16 (BD Biosciences), anti-CD33 (BioLegend, San Diego, CA, USA), anti-CD206 (Miltenyi Biotec, Bergisch Gladbach, Germany), and allophycocyanin-conjugated anti-HLA-DR (BioLegend) (Supplementary Table 5).

Post incubation, erythrocytes were lysed using fluorescence activated cell sorting lysing solution (BD Biosciences), washed twice with 1 × phosphate-buffered saline, and fixed with BD CellFix (BD Biosciences). A total of 10,000–50,000 cells were counted using a BD FACSCalibur flow cytometer and analyzed using Cellquest™ software (BD Biosciences). Each event was recorded in a forward scatter (FSC)/side scatter (SSC) plot that depicted lymphocytes, monocytes, and granulocytes according to their low, medium, or high forward scattering, complexity, and granularity. The gating strategy to identify lymphocytes, monocytes and granulocytes was performed by forward (FSC) and side scatter (SSC) analysis. Monocytes presented with larger size and moderate granularity as compared to lymphocytes and granulocytes. The correctness of the monocyte gate was further proven by the expression of HLA-DR and CD33. The confirmed monocyte gate was then subjected to CD45^+^CD14^+^ expression analysis. On the basis of co-expression intensities for CD14 and CD16, monocytes were further distinguished as CD14^+^CD16^+^, CD14^+^CD16^–^, or CD14^–^CD16^+^ subsets (Supplementary Figure 1).

### Statistical analysis

In this study, continuous variables such as age, BMI, and monocyte percentages are shown as median with interquartile range. Comparisons of parameters like age, BMI, and cell surface markers expression between groups were performed using the Mann-Whitney *U*-test or Kruskal–Wallis non-parametric test. Categorical data (e.g., sex and disease distribution) were tested with either the Chi square test or Fisher’s exact test. The Spearman rank correlation test was used to assess the association between variables and the strength of relationship was rated by absolute magnitude of the Spearman correlation coefficient (r) that ranges from negligible (0.00–0.10), weak (0.10–0.39), moderate (0.40–0.69), strong (0.70–0.89), to very strong (0.90–1.00) (19).

Univariate statistical analysis was performed with GraphPad Prism version 8.0 software.^1^ Statistical significance in all tests is defined as a *p*-value < 0.05. We also computed Cohen’s d (*d*_*s*_) to describe the standardized mean difference of a cell population amongst patients with or without diabetes, obesity, and sepsis. Positive or negative *d*_*s*_ values were interpreted as none (*d*_*s*_ ≤ 0.1 or *d*_*s*_ ≥ −0.1), small (*d*_*s*_ ≤ 0.4 or *d*_*s*_ ≥ −0.4), moderate (*d*_*s*_ ≤ 0.7 or *d*_*s*_ ≥ −0.7), or large (*d*_*s*_ ≤ 1 or *d*_*s*_ ≥ −1) effects on a cell population (20, 21).

## Results

Baseline characteristics for all patients are shown in Supplementary Table 1. The median (25th percentile, 75th percentile) age and BMI were 66 (55, 75) years and 28 (25, 33) kg/m^2^, respectively. Gender ratio, age and BMI did not significantly differ between the non-diabetic and diabetic individuals.

### Distribution of septic severity status in patients with obesity and diabetes mellitus

Specimens are broadly categorized as non-diabetic (*n* = 154) and diabetic (*n* = 81) in Table 1. In the non-diabetic group, 27 patients suffered from sepsis (16 non-obese and 11 obese) and 11 experienced septic shock (10 non-obese and 1 obese). Likewise, in the diabetic group, 22 individuals presented with sepsis (9 non-obese and 13 obese) and 15 manifested septic shock (4 non-obese and 11 obese). Within the diabetic group, the frequency for septic shock was lower in non-obese (*n* = 4) vs. obese individuals (*n* = 13; *p* > 0.05). Compared to the non-diabetic and obese subgroup, the incidence for septic shock (*n* = 1) was higher in patients with diabetes and obesity (*n* = 13; *p* < 0.05). The distribution of age, gender, and BMI are given in Supplementary Table 1.

**TABLE 1:** The following table shows the distribution of patients due to the clinical conditions on ICU (non-septic, septic, and septic shock) and how the groups devide up by obesity diabetes or both.

All patients (*N* = 235)	Non-sepsis *N* = 160 (%)	Sepsis *N* = 49 (%)	Septic shock *N* = 26 (%)
Non-obese	81 (50.6)	16 (32.6)	10 (38.5)
Obese	35 (21.9)	11 (22.4)	1 (3.8)
Non-obese diabetic	23 (14.4)	9 (18.3)	4 (15.4)
Obese and diabetic	21 (13.1)	13 (26.5)	11 (42.3)

### Effect of sepsis severity on monocyte subsets in non-diabetic and diabetic patients

**FIGURE 1:**
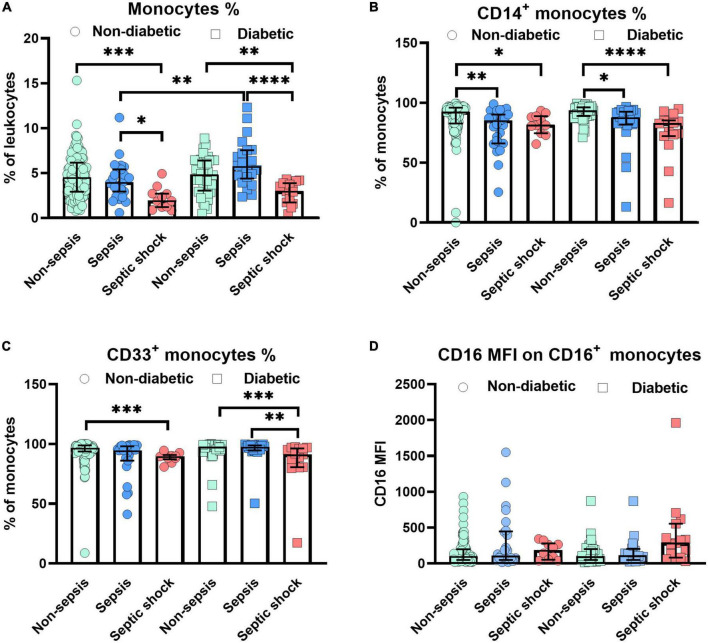
Alterations of monocytes in non-diabetic/diabetic patients with distinct degrees of sepsis severity. Distribution changes in the percentage of monocytes among the total leukocyte population **(A)** and the percentage of CD14^+^ monocytes **(B)** and CD33^+^ monocytes among the monocyte population **(C)**. **(D)** Surface phenotype expression of CD16 on positive monocytes determined by mean fluorescence intensity (MFI). Data are presented by scatter with bar plots. Results are medians with interquartile range. Kruskal–Wallis test and Mann–Whitney *U*-test were, respectively, used to analyze the differences among three groups and two groups. Significant difference is marked by *p < 0.05, **p < 0.01, ***p < 0.001, and ****p < 0.0001.

**FIGURE 2:**
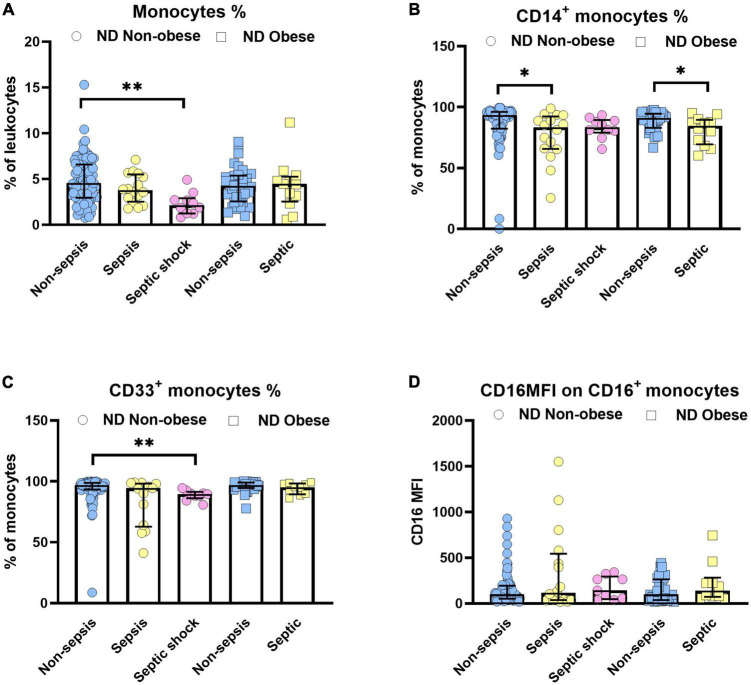
Influence of obesity on monocytes in non-diabetic (ND) patients with or without sepsis status. Distribution changes in the percentage of monocytes among the total leukocyte population **(A)** and the percentage of CD14^+^ monocytes **(B)** and CD33^+^ monocytes among the monocyte population **(C)**. **(D)** Surface phenotype expression of CD16 on positive monocytes determined by mean fluorescence intensity (MFI). Data are presented by scatter with bar plots. Due to the small sample size, the obese group was not divided into sepsis (*n* = 11) and septic shock (*n* = 1) subgroups, rather presented as a combined “septic” group. Results are medians with interquartile range. Kruskal–Wallis test and Mann–Whitney *U*-test were, respectively, used to analyze the differences among three groups and two groups. Significant difference is marked by *p < 0.05 and **p < 0.01.

**FIGURE 3:**
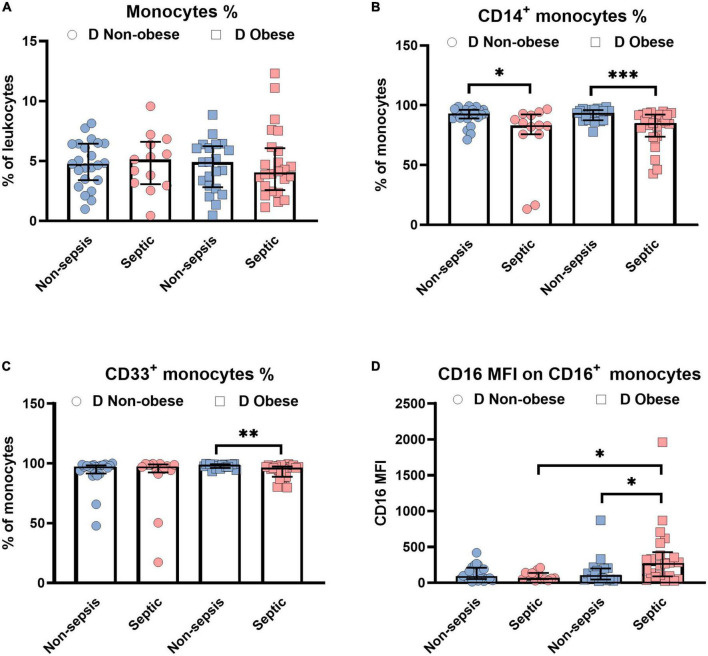
Influence of obesity on monocytes in diabetic **(D)** patients with or without sepsis status. Distribution changes in the percentage of monocytes among the total leukocyte population **(A)** and the percentage of CD14^+^ monocytes **(B)** and CD33^+^ monocytes among the monocyte population **(C)**. **(D)** Surface phenotype expression of CD16 on positive monocytes determined by mean fluorescence intensity (MFI). Data are presented by scatter with bar plots. Due to the small sample sizes, the non-obese and obese groups were not divided into sepsis (non-obese, *n* = 9; obese, *n* = 13) and septic shock (non-obese, *n* = 4; obese, *n* = 11) subgroups, rather presented as a combined “septic” group. Results are medians with interquartile range. Mann-Whitney *U*-test was used to analyze the differences between two groups. Significant difference is marked by *p < 0.05, **p < 0.01, and ***p < 0.001.

The current analysis focuses on surface markers expressed by peripheral blood derived monocytes. In addition to the endotoxin binding molecule CD14 and the immune complex receptor CD16, we also studied CD33, a silencing molecule (Siglec-3) which is linked to the maturity and phagocytic activity. The scavenger receptors, CD163 and CD206 were also tested since these identify the anti-inflammatory phenotype of M2 macrophages to be distinguished from pro-inflammatory M1 subsets. Figure 1 shows the relative distribution of monocyte subpopulations in patients with and without diabetes in patients without sepsis, sepsis and septic shock. Monocytes expressing CD14^+^ (classical) or CD33^+^, as well as the CD16 expression density on monocytes revealed significant differences in the median expression levels marked by *p*-values (Figure 1). We further compared non-diabetic patients with and without obesity (Figure 2). Obese patients differed from non-obese patients in that their classical monocytes were more diminished with the manifestation of sepsis/septic shock, but the diminution of CD33^+^ monocytes was similar. Figure 3 compares monocyte sub-populations of non-obese and obese diabetic patients with and without sepsis. Only obese patients with sepsis presented with a significant decline in their total amount of CD14^+^ and CD33^+^ monocytes when compared with the non-sepsis group. In addition, obese sepsis patients displayed greater up-regulated CD16 expression densities than non-obese sepsis patients.

According to the Cohen’s effect size scale, the decline in percentage of the CD14^+^ monocytes and the CD33^+^ monocytes constituted the most significant parameters in obese patients suffering from sepsis including septic shock (Supplementary Table 6). In the absence of diabetes and obesity, the negative impact of sepsis including septic shock on CD14^+^ and CD33^+^ monocytes were more moderate (*d*_*s*_ = −0.3 to −0.8) when compared with obese diabetic patients (*d*_*s*_ = −1.0). The down modulation was large in septic patients with diabetes for CD14^+^% (*d*_*s*_ = −0.7 to −1.4) and septic individuals with obesity for CD14^+^% (*d*_*s*_ = −0.9). Additionally, the relative amounts of CD33^+^% (*d*_*s*_ = −1.0) were adversely impacted in septic patients with diabetes and obesity (Supplementary Table 6). Like the percentage of CD14^+^ monocytes (Supplementary Table 6), diabetes in septic patients without obesity negatively impacted the CD14^+^CD16^–^% (*d*_*s*_ = −1.1). Lastly, the presence of diabetes led to the up-regulation of the percentage of the CD14^–^CD16^+^ monocyte subset (*d*_*s*_ = 0.4) in septic patients with obesity (Supplementary Table 6).

In summary, increasing pro-inflammation with the transition of sepsis to septic shock appears to be associated with lower CD14^+^ and CD33^+^ and up-regulation of CD16, particularly in obese patients. To prove this hypothesis, we further tested CD163, the marker for anti-inflammatory macrophages. According to Supplementary Table 2, CD163 densities were higher in non-diabetic patients with sepsis as compared to non-sepsis (*p* > 0.05), whereas diabetic patients revealed a significant upregulation of CD163 densities only when comparing non-sepsis and sepsis cases with those manifesting septic shock (*p* = 0.002). Quantitative amounts of CD163^+^ monocytes were not different between non-diabetic patient groups neither with nor without obesity (Supplementary Table 2). Thus, diabetic patients with sepsis are more likely to activate the anti-inflammatory CD163. These changes did not correspond with alterations of HLA-DR expression densities (Supplementary Tables 2, 3).

**FIGURE 4:**
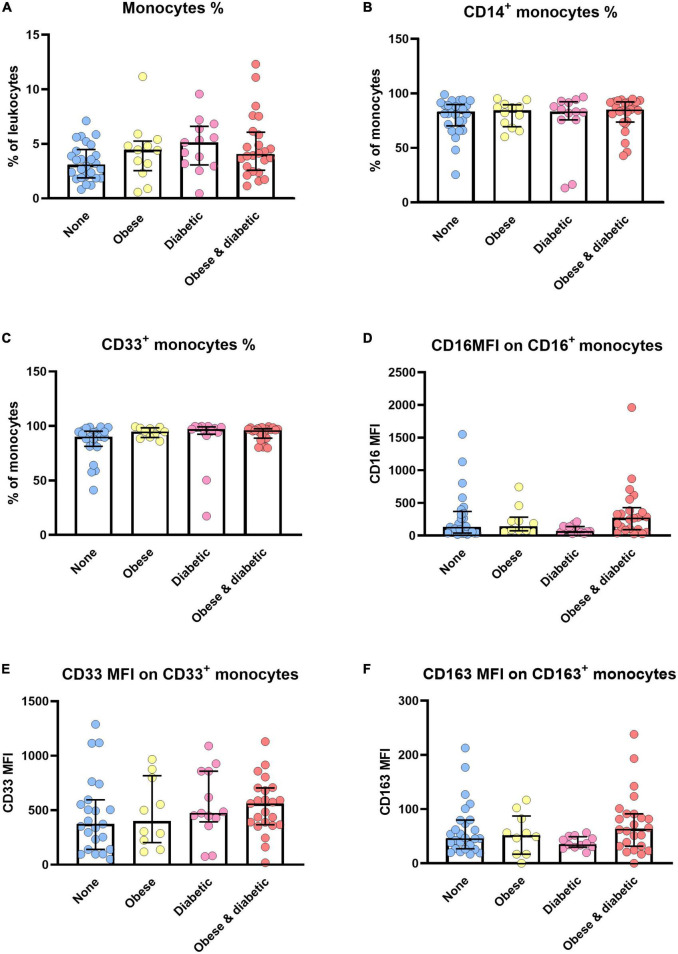
Monocyte alterations in sepsis/septic shock patients with and without obesity, diabetes or both. Distribution changes in the percentage of monocytes among the total leukocyte population **(A)** and the percentage of CD14^+^ monocytes **(B)** and CD33^+^ monocytes among the monocyte population **(C)**. **(D)** Surface phenotype expression of CD16, **(E)** CD33 and **(F)** CD163 on positive monocytes determined by mean fluorescence intensity (MFI). Data are presented by scatter with bar plots. Results are medians with interquartile range. Kruskal-Wallis test were respectively used to analyze the differences among four groups. Significant difference is marked by *p < 0.05.

Figure 4 summarizes phenotype alterations of monocyte subsets among patients with and without obesity and with and without diabetes. Accordingly, the expression density of CD16 ist significantly different between diabetic patients and obese and diabetic patients. Here, we combined patients with sepsis and septic shock (Figure 4D). The same trend was observed when analyzing CD163 expression densities (Figure 4F).

### The impact of age on monocyte subpopulations of diabetic patients with sepsis

Since monocytes are indicators of aging of the immune system, we also checked the correlation between monocyte subtypes and age in diabetic patients with sepsis/septic shock. Notably, old patients tended to have lower HLA-DR expression densities (*p* = 0.004, *r* = −0.63).

## Discussion

Poor clinical outcome in patients who are critically ill with sepsis has been attributed to monocytic deactivation, diminished expression of major histocompatibility class II molecules, and an impaired immune system (1, 2, 4). We here focused on monocyte subsets in obese or diabetic patients treated by ICU. We demonstrated the modulation of monocyte subsets in obese and type 2 diabetes individuals experiencing sepsis (Figures 1–3). In all patients developing sepsis and septic shock, monocytes as well as CD14^+^, CD33^+^, CD14^+^CD16^–^, and CD14^–^CD16^+^ were significantly reduced (*p* < 0.05) (Figures 1–3), which is in line with the previous report (22). The negative impact on the above-mentioned cell populations in septic patients without diabetes or obesity was moderate (*d*_*s*_ = −0.3 to −0.9, Supplementary Table 6). However, this became large (*d*_*s*_ ≥ −0.9) under the combined influence of diabetes and obesity in such patients (Supplementary Table 6).

Regarding sepsis and diabetes, the CD14^+^CD16^–^ subset was affected most in non-obese but not in obese patients (Supplementary Table 4). Interestingly, a similar decrease in the CD14^+^CD16^–^ subset was observed in non-obese individuals with severe COVID-19 and type 2 diabetes (23).

Furthermore, our data confirmed previous reports in that circulating CD14 expression densities were elevated in non-infected diabetes as well as in sepsis patients (24, 25). We observed similar changes of CD14 expression on CD14^+^CD16^–^ monocytes in non-diabetic patients, which displayed higher levels in the non-obese septic subgroup relative to the obese septic subgroup (Supplementary Table 3).

By contrast, another study reported a decreased surface CD14 expression in sepsis/septic shock patients (26). Although CD14 expression was trend wise lower on sepsis progression, we found no significant differences within the groups of non-sepsis, sepsis, and septic shock with or without diabetes (Supplementary Table 2). However, among non-diabetic sepsis patients, CD14 levels on the CD14^+^CD16^–^ subset were decreased when compared to diabetic patients (Supplementary Table 2). This observation may be related to the fact that CD14 expression was associated with insulin sensitivity and the occurrence of obesity and diabetes as a state of chronic low grade inflammation (27). Similarly, under non-diabetic conditions, non-obese septic patients displayed a relatively increased CD14 expression on the classical CD14^+^CD16^–^ subset in contrast to obese septic patients (Supplementary Table 3). This observation may reflect a compensatory response to enhanced inflammation and a higher capacity to activate Toll like receptor (TLR)-4 in the context of pathogen defense (28). Recent evidence demonstrated that metabolic reprogramming may also contribute to the development of classical monocyte dysfunction during sepsis (29).

### Intermediate monocyte activation in sepsis and relationship with the body mass index

Distinct from classical monocytes, a minority of CD16^+^ monocytes characterized by high HLA-DR expression, high amounts of pro-inflammatory cytokines, high Fcγ-R mediated phagocytosis, and high antigen-presenting cell activity, but low to absent interleukin (IL)-10 secretion, were identified within the subsets of CD14^+^CD16^+^ and CD14^–/dim^CD16^+^ monocytes (6, 30). Generally, CD14^+^CD16^+^ monocyte expansion was found in various diseases including obesity, diabetes, and sepsis compared with healthy controls (6, 13, 31). Results are in line with our critically ill patients. Still, the percentage of the CD14^+^CD16^+^ subset was negatively associated with BMI in non-diabetic septic patients and diabetic septic patients (Supplementary Tables 3, 4). Similarly, the association of age with CD14^+^CD16^+^ monocytes has been reported in healthy donors (32). All changes related to age of our patients support their potential role for “immunosenescence” and age-related adaptive immunity by monocyte reprogramming (32).

### Non-classical monocyte activation relies on sepsis in obese and/or diabetic patients

As a fraction of CD16^+^ monocytes, the non-classical CD14^–/dim^CD16^+^ population has been characterized to be involved in complement and Fc gamma-mediated phagocytosis and adhesion. This cell type is active in patrolling and may play a role in vascular diseases (7, 9). Unlike the other two CD14^+^ subpopulations, we found a higher proportion of the non-classical subset in all sepsis patients with diabetes compared to non-diabetic patients (Supplementary Table 2). Increased percentages of CD14^–^CD16^+^ monocytes were found in septic patients with both, obesity and diabetes (Supplementary Table 4), which is consistent with previous reports (31, 33). Obesity affected both, the distribution and phenotypic expression of monocyte subsets in non-diabetic and diabetic patients. Of note, the CD14^–^CD16^+^ population was increased in septic patients with diabetes and obesity but not in non-diabetic and non-obese septic patients (Supplementary Tables 3, 4). These findings imply a selective influence of diabetes on CD14^–^CD16^+^ monocytes in states of immune insufficiency and sepsis (34). IL-10 may lead to the expansion of CD16^+^ monocytes, thus diabetes treatment could efficiently combat pro-inflammation by CD16 + monocytes under septic conditions. This hypothesis clearly requires further investigations.

While our observations in adult patients were supported by another study (22), neonates and pediatric cases with sepsis were characterized by an increased subset of non-classical monocytes (35), supporting age-related effects. Inflammatory conditions preceding sepsis appear to be key for intermediate, CD14^+^CD16^+^ and non-classical, CD14^–^CD16^+^ monocyte up-regulation (36). Moreover, low CD14^dim^/CD16^pos^/CD45^pos^ patrolling monocytes in sepsis were linked to immunosuppression and inferior survival (37).

### CD16 up-regulation is particularly involved in sepsis with obesity and diabetes

Despite similar changes of the monocyte subsets including CD14^+^ and CD33^+^ in obese septic patients with diabetes, CD16 expression densities were higher in sepsis patients than in the non-sepsis group (Figure 3D). According to Shalova et al. CD16 could function as a key regulator of TIR-domain-containing adapter-inducing interferon-β -dependent TLR4 signaling in monocytes by driving negative regulators of the MyD88-dependent TLR4 pathway (38). As a consequence, CD16^+^ monocytes appear to be involved in this endotoxin-tolerant and immunosuppressive pathway. Results are further supported by a rodent sepsis model (39). Monoclonal antibody (mAb)-induced CD16 inhibition also increased the survival of mice with high-grade sepsis (39). The selective influence by TNF from CD16^+^ monocytes (40) to the induction of endotoxin tolerance in the later phase of the disease (38) appears to be highly relevant.

### CD33 modulation patterns associated with sepsis severity in diabetic patients

CD33 is a myeloid-specific member of the sialic acid-binding receptor family and is expressed at higher levels on the CD14^+^CD16^+^ and CD14^+^CD16^–^ subpopulations compared to the CD14^–^CD16^+^ subpopulation (13, 30, 41), and augmented inflammatory cytokine production (41). It was reported to function as an inhibitory receptor for inflammatory production, such as IL-1β, IL-8, and TNF-α, because blockade by anti-CD33 mAb or silencing by small interfering RNA led to increased pro-inflammatory cytokine release (41–43). Lower CD33 also contributes to the diabetes-related inflammation profile with increased levels of TNF-α, IL-8, and IL-12p70 (44). Hyperglycemic conditions in cultured monocytes from healthy donors showed significantly decreased CD33 protein and mRNA expression, but increased spontaneous TNF-α secretion and suppressor of cytokine signaling protein-3 mRNA expression (44). Hyperglycemia resulted in relatively increased percentages of CD33^low^ cells (mainly known as CD14^–^CD16^+^) and decreased amounts of CD33^high^ populations (including CD14^+^CD16^–^ and CD14^+^CD16^+^) (13, 30, 44). However, the role of CD33 in sepsis with and without diabetes, including the relevance of hyperglycemia, remains ill defined (45–47). We observed that the percentage of CD33^+^ monocytes was significantly diminished in septic shock patients with or without diabetes (Figure 1C). Moreover, the proportion of CD33^+^ monocytes was even lower in septic shock when compared to sepsis in diabetic patients (Figure 1C). Non-diabetic patients exhibited lower levels of this population and CD33 expression densities (Figure 1C and Supplementary Table 2). These changes imply that the pro-inflammatory response to an acute infection at an early stage may be deficient in diabetic patients.

### CD163 upregulation correlated with sepsis with obesity and diabetes

CD163, CD206, and arginase-1 (Arg-1) act as makers of an alternative activation of monocytes/macrophages (M2, anti-inflammatory phenotype) (48), and play a crucial role in chronic inflammation of obesity-related metabolic diseases (49, 50). CD163 was found to be expressed mainly on the classical pro-inflammatory monocytes (CD14^+^CD16^–^) as compared with the non-classical subset (CD14^–/dim^CD16^+^) (9). Decreased percentages of CD163^+^CD14^+^ monocytes and increased monocytic CD163 were observed in type 2 diabetes and obesity (49, 50), as well as in sepsis patients (51). In agreement with previous results, our data showed that CD163 expression was greatly higher in obese and diabetic with septic shock as well as obese when compared with non-obese patients (Supplementary Table 4 and Figure 4F). The increased CD163 expression implies, that monocytes might acquire the alternatively activated anti-inflammatory phenotypes during the manifestation of sepsis, even though a decreased production of inflammatory cytokines was observed in LPS-tolerant human monocytes, regardless of the CD163 or CD206 expression (51). Monocyte polarization to anti-inflammatory phenotypes and endotoxin tolerance are believed to be responsible for sepsis-induced immune insufficiency and may explain higher mortality (52).

In the current study, we did not find significant differences regarding other anti-inflammatory monocyte phenotypes, including CD206 and Arg-1, although CD206 had a weak expression in all critically ill patients, which is supported by previous observations (53). No BMI-related changes were observed among non-septic groups, but the scavenger receptor CD206 correlated with BMI in diabetic septic patients which may indicate accelerated immunosenescence in diabetic patients (54) (Supplementary Table 4). This interesting observation should be subject to confirmation in a larger observational study.

### Study strengths and limitations

In the present study, it is inevitable that there were confounding factors and limitations. Therefore, some issues should be considered when assessing the results, such as dynamic monitoring of the alteration of sensitive and special immune indicators and clinical parameters, including the assessment of glucose control and survival. Moreover, the evaluation of distinct comorbidities and complications, sites of infection, types of pathogens, antibiotic therapy, hypoglycemic agents, and comprehensive treatment strategies might help to understand individual variations of a given patient and subgroups. More importantly, more detailed monocyte classifications could be performed using single-cell RNA sequencing approaches (55). A most important limitation of the current study is the low number of patients in defined subgroups such as non-diabetic, obese patients. For a more definitive conclusion, a new observational study needs to be initiated to specifically address the difference in monocyte guided immunity of diabetic patients with and without obesity.

Despite these limitations, we believe that the findings of our study contribute to the comprehensive knowledge of monocyte subset modulation involved in the complex context of metabolic disease (obesity and diabetes) in critically ill patients. Furthermore, findings from our study may provide a supportive reference for further investigations.

## Conclusion

The relative amounts of circulating CD14-positive monocytes were clearly affected in patients during the transition from non-sepsis to sepsis and subsequently to septic shock independently of diabetes and obesity. Although acute inflammation affected the phenotypic modulation of monocytes in obese and/or diabetic patients during sepsis, pre-existing disease may be relevant for the up-regulation of CD16 in obesity and diabetes. Targeting this antigen as well as the immunosuppressive CD33 could be promising preventative targets in obese and diabetic patients at a high risk for sepsis and mortality.

Herein, our study included only pre-COVID-19 specimens, but may be applied to current studies of patients with the severe sepsis based on acute virus infections such as SARS-CoV2 in obese patients (56, 57).

## Data availability statement

The original contributions presented in this study are included in the article/Supplementary material, further inquiries can be directed to the corresponding author.

## Ethics statement

The studies involving human participants were reviewed and approved by the Ulm University Ethics Committee. The patients/participants provided their written informed consent to participate in this study.

## Author contributions

EMS, DN, and KG wrote the manuscript. EMS designed the laboratory experiments of the study. BM supervised the statistical work-up. BS, EB, and HB performed the clinical study and supervised patients to be selected for the analysis. MW screened and collected all patients’ data sets and recordings from ICU data sets. JS supported graphical and statistical analysis. DN conducted the lab experiments and performed the statistical analysis. KG and CL contributed to defined data management and analysis. All authors revised the manuscript, read, and approved the final manuscript.

## Footnotes

1
https://GraphPad.com

## Supplementary material

Supplementary Figure 1 CD14 and CD16 staining of peripheral blood monocytes in one patient specimen. **(A)** Identification of lymphocytes (Rl gate), monocytes (R2 gate), and granulocytes (R3 gate) displayed in a representative FSC/SSC dot plot. **(B)** Staining by FITC-conjugated anti-CD 14 and PE-conjugated anti-CD 16 antibodies. Monocytes were gated based on their light scatter profiles, and subsets identified by the fluorescence gates of CD14-FITC and CD16-PE: CD14^–^CD16^–^ monocytes (lower right gate), CD14^+^CD16^–^ monocytes (upper right gate), and CD14^–^CD16^+^ monocytes (upper left gate), with respective percentage in the box.
