# Supplementary material for: Monocyte subtype expression patterns in septic patients with diabetes are distinct from patterns observed in obese patients
Source: Front Med (Lausanne). 2023 Jan 5;9:1026298. doi: 10.3389/fmed.2022.1026298 (PMC9849690; doi:10.3389/fmed.2022.1026298)
Supplement: Supplementary file 1 [file Table_1.docx]

Table S1: Patients’ characteristics

| Parameter | All  (n=235) |  | Non-diabetic | | |  | Diabetic | | |
| --- | --- | --- | --- | --- | --- | --- | --- | --- | --- |
|  |  |  | All  (n=154) | Non-obese  (n=107) | Obese  (n=47) |  | All  (n=81) | Non-obese  (n=36) | Obese  (n=45) |
| Age (years) | 66 (55, 75) |  | 62 (54, 75) | 63 (53, 76) | 61 (54, 71) |  | 70 (60, 77) | 73 (63, 78) | 66 (54, 74) |
| Male/Female, n | 160/75 |  | 106/48 | 73/34 | 33/14 |  | 54/27 | 30/6 | 24/21 |
| BMI (kg/m^2^) | 28 (25, 33) |  | 28 (24, 31) | 25 (24, 28) | 34 (31, 37) |  | 31 (27, 36) | 26 (25, 28) | 35 (31, 39) |
| Sepsis status, n |  |  |  |  |  |  |  |  |  |
| Non-sepsis | 160 |  | 116 | 81 | 35 |  | 44 | 23 | 21 |
| Sepsis | 49 |  | 27 | 16 | 11 |  | 22 | 9 | 13 |
| Septic shock | 26 |  | 11 | 10 | 1 |  | 15 | 4 | 11 |

Results are expressed as median (25th percentile, 75th percentile). n: number; BMI: body mass index.
