# Supplementary material for: Monocyte subtype expression patterns in septic patients with diabetes are distinct from patterns observed in obese patients
Source: Front Med (Lausanne). 2023 Jan 5;9:1026298. doi: 10.3389/fmed.2022.1026298 (PMC9849690; doi:10.3389/fmed.2022.1026298)
Supplement: Supplementary file 2 [file Table_2.docx]

Table S2: Comparison of blood monocyte subsets in non-diabetic and diabetic patients with/without sepsis

| **Parameter^⁜^** | **Non-diabetic** | | | ***p*-value** | **Diabetic** | | | ***p*-value** |
| --- | --- | --- | --- | --- | --- | --- | --- | --- |
|  | **Non-sepsis**  **(n=116)** | **Sepsis**  **(n=27)** | **Septic shock**  **(n=11)** |  | **Non-sepsis**  **(n=44)** | **Sepsis**  **(n=22)** | **Septic shock**  **(n=15)** |  |
| **Age (years)** | 63 (54, 75) | 57 (51, 68) | 66 (53, 76) | 0.221 | 68 (60, 77) | 73 (60, 80) **※※** | 64 (53, 73) | 0.283 |
| **Male/Female, n** | 79/37 | 20/7 | 8/3 | 0.808 | 30/14 | 16/5 | 9/6 | 0.582 |
| **BMI (kg/m^2^)** | 28 (24, 31) | 28 (24, 33) | 25 (24, 26) | 0.138 | 29 (25, 34) | 31 (28, 35) | 36 (29, 43) **§§§** | 0.057 |
| **Non-obese/Obese, n** | 81/35 | 16/11 | 10/1 | 0.156 | 23/21 | 9/13 | 4/11 | 0.210 |
| **Monocytes%^a^** | 4.5 (2.9, 6.2) | 3.9 (2.9, 5.4) | 1.9 (1.2, 2.7) *****#** | **0.001** | 4.8 (3.0, 6.4) | 5.7 (4.4, 7.5) **※※** | 3.0 (1.7, 3.9) ****###****#** | **0.0001** |
| **A:****CD14^+^CD16^+^%^b^** | 28 (7.8, 60) | 25 (12, 62) | 27 (20, 40) | 0.948 | 35 (9.8, 61) | 38 (14, 58) | 30 (17, 47) | 0.726 |
| **B:****CD14^+^CD16^−^%^b^** | 52 (30, 80) | 42 (19, 65) | 50 (41, 74) | 0.223 | 55 (28, 84) | 40 (25, 62) | 45 (22, 60) | 0.222 |
| **C:****CD14^−^CD16^+^%^b^** | 1.4 (0.57, 3.0) | 0.88 (0.58, 3.2) | 2.6 (0.0, 6.1) | \|  \| \| --- \|   0.916 | 1.8 (1.0, 3.5) | 3.0 (1.3, 4.7) **※** | 1.9 (0.4, 7.1) | 0.462 |
| **CD14 MFI-A** | 589 (375, 743) | 495 (394, 650) | 502 (363, 720) | 0.427 | 551 (394, 719) | 526 (381, 639) | 348 (228, 744) | 0.414 |
| **CD14 MFI-B** | 420 (299, 546) | 323 (149, 390) ***** | 374 (267, 504) | **0.045** | 398 (288, 651) | 309 (259, 423) | 414 (195, 521) | 0.235 |
| **CD14 MFI-C** | 10 (6.5, 13) | 10 (6.4, 13) | 7.4 (0.0, 13) | 0.587 | 10 (8.9, 12) | 9.7 (7.9, 11) | 11 (8.8, 21) | 0.385 |
| **CD16 MFI-A** | 93 (47, 201) | 96 (48, 457) | 166 (55, 268) | 0.317 | 88 (44, 181) | 88 (42, 136) | 171 (47, 413) | 0.428 |
| **CD16 MFI-B** | 8.4 (5.9, 13) | 8.3 (6.7, 12) | 12 (7.2, 15) | 0.189 | 9.8 (6.0, 13) | 9.8 (6.6, 13) | 7.6 (5.6, 15) | 0.875 |
| **CD16 MFI-C** | 173 (46, 429) | 137 (25, 418) | 45 (0.0, 365) | 0.240 | 171 (72, 386) | 148 (33, 423) | 188 (76, 588) | 0.738 |
| **CD14^+^%^b^** | 97 (94, 99) | 85 (66, 90) ****** | 81 (75, 89) ***** | **0.0002** | 93 (89, 96) | 88 (82, 93) ***※** | 83 (72, 85) ******** | **0.0001** |
| **CD14 MFI** | 557 (360, 886) | 402 (224, 740) | 443 (325, 586) | 0.122 | 540 (411, 719) | 450 (361, 647) | 428 (250, 715) | 0.378 |
| **CD16^+^%^b^** | 67 (37, 83) | 48 (32, 70) | 27 (25, 41) | 0.991 | 37 (14, 66) | 45 (21, 62) | 31 (26, 41) | 0.556 |
| **CD16 MFI** | 195 (99, 338) | 181 (129, 367) | 183 (53, 279) | 0.423 | 95 (50, 202) | 110 (50, 203) | 287 (82, 556) ***#** | **0.014** |
| **CD33^+^%^b^** | 97 (94, 99) | 95 (86, 98) | 90 (87, 91) ******* | **0.0002** | 98 (95, 99) | 98 (95, 99) | 91 (81, 96) *****##** | **0.0007** |
| **CD33 MFI** | 557 (360,886) | 402 (224, 740) | 318 (98, 693) | 0.065 | 578 (483, 791) | 562 (410, 858) | 448 (162, 705) | 0.174 |
| **HLA-DR^+^%^b^** | 67 (37, 83) | 48 (32, 70) | 31 (26, 60) | 0.092 | 78 (35, 89) | 48 (18, 73) | 64 (56, 74) | 0.721 |
| **HLA-DR MFI** | 195 (99, 338) | 181 (129, 367) | 233 (116, 391) | 0.938 | 160 (97, 435) | 157 (68, 344) | 314 (201, 485) | 0.236 |
| **Ratio A%/B%** | 0.5 (0.09, 1.7) | 0.76 (0.17, 3.6) | 0.5 (0.27, 0.97) | \|  \| \| --- \|   0.659 | 0.63 (0.12, 2.1) | 0.97 (0.29, 2.2) | 0.67 (0.25, 1.3) | 0.460 |
| **CD163^+^%^b^** | 33 (8.1, 67) | 10 (4.8, 61) | 24 (8.3, 45) | 0.159 | 23 (2.3, 47) | 22 (7.2, 62) | 31 (8.7, 53) | 0.479 |
| **CD163 MFI** | 39 (28, 55) | 49 (31, 81) ****** | 47 (20, 80) | 0.254 | 32 (25, 46) | 34 (26, 56) | 71 (39, 101) ****** | **0.002** |
| **CD206^+^%^b^** | 6.3 (2.6, 21) | 3.3 (1.3, 15) | 4.6 (3.3, 12) | 0.169 | 5.4 (2.0, 18) | 10 (3.0, 27) | 5.8 (3.0, 16) | 0.476 |
| **CD206 MFI** | 44 (28, 73) | 37 (26, 91) | 48 (26, 79) | 0.851 | 31 (23, 87) | 35 (27, 70) | 105 (34, 310) | 0.081 |
| **Arg-1^+^%^b^** | 4.2 (2.1, 13) | 4.0 (2.8, 7.4) | 15 (3.6, 18) | 0.106 | 4.1 (3.1, 9.3) | 3.4 (1.6, 5.5) | 3.2 (1.3, 9.6) | 0.481 |
| **Arg-1 MFI** | 37 (27, 62) | 29 (25, 60) | 28 (25, 47) | 0.310 | 33 (23, 54) | 32 (24, 52) | 45 (35, 85) | 0.052 |

**⁜**% a: monocytes among all leukocytes, b: monocyte subset in total monocytes; n: number; BMI: body mass index; MFI: mean fluorescence intensity.

Monocyte subsets: CD14^+^CD16^+^ monocytes (A), CD14^+^CD16^−^ monocytes (B), CD14^−^CD16^+^ monocytes (C).

Results are medians (25th percentile, 75th percentile). The *p* values are shown for comparisons among the three groups in non-diabetic or diabetic patients, respectively.

**p* < 0.05, ***p* < 0.01, ****p* < 0.001, *****p* < 0.0001, vs. non-sepsis of non-diabetic or diabetic patients, respectively;

#*p* < 0.05, ##*p* < 0.01, vs. sepsis group of non-diabetic or diabetic patients, respectively;

※*p* < 0.05, *p* < 0.01, vs. sepsis subgroup of non-diabetic patients;

§§§*p* < 0.001, vs. septic shock subgroup of non-diabetic patients.
