# Supplementary material for: Monocyte subtype expression patterns in septic patients with diabetes are distinct from patterns observed in obese patients
Source: Front Med (Lausanne). 2023 Jan 5;9:1026298. doi: 10.3389/fmed.2022.1026298 (PMC9849690; doi:10.3389/fmed.2022.1026298)
Supplement: Supplementary file 3 [file Table_3.docx]

Table S3: Comparison of blood monocyte subsets in non-diabetic patients with/without obesity in distinct sepsis states

| **Parameter^⁜^** | **Non-diabetic** | | | | | |  |
| --- | --- | --- | --- | --- | --- | --- | --- |
|  | **Non-obese** | | | ***p*-value** | **Obese** | | ***p*-value** |
|  | Non-sepsis  (n=81) | Sepsis  (n=16) | Septic shock  (n=10) |  | Non-sepsis  (n=35) | Septic  (n=12) |  |
| **Age (years)** | 65 (55, 78) | 57 (51, 68) | 67 (53, 77) | 0.439 | 62 (54, 71) | 59 (41, 71) | 0.361 |
| **Male/Female, n** | 57/24 | 10/6 | 7/3 | 0.822 | 23/12 | 11/1 | 0.136 |
| **BMI (kg/m^2^)** | 25 (24, 28) | 25 (21, 28) | 25 (23, 26) | 0.480 | 34 (31, 37) **††††** | 34 (32, 46) **§§§§** | 0.957 |
| **Monocytes%^a^** | 4.5 (3.0, 6.6) | 3.7 (2.5, 5.5) | 2.1 (1.2, 2.9) ****** | **0.003** | 4.2 (2.6, 5.4) | 4.4 (2.5, 5.3) | 0.843 |
| **A:CD14^+^CD16^+^%^b^** | 23 (5.8, 57) | 32 (16, 61) | 26 (18, 42) | 0.588 | 36 (12, 65) | 18 (5.9, 74) | 0.447 |
| **B:CD14^+^CD16^−^%^b^** | 55 (32, 84) | 39 (22, 57) | 55 (39, 74) | 0.082 | 52 (28, 74) | 59 (18, 70) | 0.952 |
| **C:CD14^−^CD16^+^%^b^** | 1.3 (0.47, 3.0) | 0.84 (0.25, 3.2) | 2.9 (0.23, 6.1) | \|  \| \| --- \|   0.799 | 1.6 (0.64, 3.0) | 0.86 (0.58, 4.5) | \|  \| \| --- \|   0.301 |
| **CD14 MFI-A** | 571 (378, 738) | 483 (304, 556) | 485 (356, 726) | 0.312 | 606 (345, 787) | 615 (416, 776) | 0.590 |
| **CD14 MFI-B** | 430 (284, 578) | 314 (139, 363) ***** | 378 (255, 513) | **0.020** | 409 (308, 534) | 357 (220, 774) **§** | 0.716 |
| **CD14 MFI-C** | 10 (7.2, 14) | 7.3 (1.6, 11) | 11 (1.9, 14) | 0.178 | 8.2 (4.4, 12) | 12 (9.3, 24) | 0.075 |
| **CD16 MFI-A** | 98 (47, 200) | 102 (38, 501) | 107 (49, 271) | 0.760 | 84 (34, 224) | 117 (78, 290) | 0.130 |
| **CD16 MFI-B** | 8.7 (5.8, 13) | 8.3 (6.2, 12) | 10 (7.0, 15) | 0.373 | 7.9 (6.0, 12) | 11 (7.7, 17) | **0.036** |
| **CD16 MFI-C** | 161 (56, 446) | 95 (19, 588) | 45 (6.9, 390) | 0.310 | 218 (29, 407) | 169 (41, 385) | 0.833 |
| **CD14^+^ %^b^** | 93 (82, 96) | 83 (66, 92) ***** | 83 (79, 89) | **0.002** | 91 (82, 95) | 85 (69, 90) | **0.022** |
| **CD14 MFI** | 517 (402, 758) | 432 (321, 503) | 428 (307, 591) | 0.052 | 525 (377, 738) | 557 (332, 736) | >0.99 |
| **CD16^+^%^b^** | 27 (8.8, 57) | 32 (22, 63) | 29 (23, 44) | 0.187 | 39 (16, 66) | 21 (8.0, 50) | 0.340 |
| **CD16 MFI** | 98 (53, 195) | 117 (37, 544) | 142 (48, 294) | 0.854 | 97 (37, 265) | 138 (73, 283) | 0.248 |
| **CD33^+^%^b^** | 97 (93, 99) | 94 (63, 98) | 90 (86, 91) ****** | **0.001** | 97 (95, 99) | 95 (90, 98) | 0.168 |
| **CD33 MFI** | 607 (395, 940) | 449 (210, 632) | 297 (98, 834) | 0.051 | 537 (305, 700) | 402 (203, 817) | 0.640 |
| **HLA-DR^+^%^b^** | 67 (37, 85) | 56 (45, 70) | 31 (26, 60) | 0.631 | 64 (43, 79) | 32 (18, 66) | 0.965 |
| **HLA-DR MFI** | 193 (64, 329) | 181 (129, 367) | 233 (116, 391) | 0.805 | 198 (113, 532) | 185 (74, 618) | 0.248 |
| **Ratio A%/B%** | 0.44 (0.07,1.4) | 0.97 (0.42, 3.2) | 0.46 (0.24, 1.2) | \|  \| \| --- \|   0.242 | 0.78 (0.18, 2.4) | 0.28 (0.09, 7.4) | \|  \| \| --- \|   0.519 |
| **CD163^+^%^b^** | 30 (4.6, 70) | 13 (5.6, 66) | 29 (14, 50) | 0.664 | 42 (18, 57) | 6.1 (0.4, 25) **§** | **0.012** |
| **CD163 MFI** | 39 (28, 55) | 46 (29, 78) | 54 (25, 85) | 0.394 | 40 (26, 54) | 52 (17, 87) | 0.560 |
| **CD206^+^%^b^** | 6.1 (2.7, 18) | 3.8 (1.4, 17) | 4.5 (3.2, 12) | 0.406 | 7.9 (2.5, 40) | 3.3 (1.0, 22) | 0.286 |
| **CD206 MFI** | 43 (29, 72) | 37 (25, 76) | 60 (37, 81) | 0.601 | 50 (28, 77) | 35 (23, 178) | 0.788 |
| **Arg-1^+^%^b^** | 4.4 (2.1, 13) | 3.8 (2.8, 6.6) | 13 (3.6, 19) | 0.151 | 4.2 (2.1, 9.2) | 6.5 (3.0, 20) | 0.210 |
| **Arg-1 MFI** | 39 (27, 62) | 29 (24, 44) | 27 (24, 45) | 0.107 | 34 (26, 56) | 49 (25, 104) | 0.450 |

**⁜**% a: monocytes among all leukocytes, b: monocyte subset in monocytes; n: number; BMI: body mass index; MFI: mean fluorescence intensity.

Given the sample size, the obese group was only divided into non-septic and septic subgroups (combined sepsis and septic shock patients).

Monocyte subsets: CD14^+^CD16^+^ monocytes (A), CD14^+^CD16^−^ monocytes (B), CD14^−^CD16^+^monocytes (C).

Results are medians (25th percentile, 75th percentile).

The *p* values are shown for comparisons among subgroups in non-obese or obese non-diabetic patients, respectively.

**p* < 0.05, ***p* < 0.01, vs. non-sepsis subgroup of non-diabetic and non-obese patients;

††††*p* < 0.0001, vs. non-sepsis subgroup of non-diabetic and non-obese patients;

§*p* < 0.05, §§§§*p* < 0.0001, vs. septic subgroup of non-diabetic and non-obese patients.
