# Supplementary material for: Monocyte subtype expression patterns in septic patients with diabetes are distinct from patterns observed in obese patients
Source: Front Med (Lausanne). 2023 Jan 5;9:1026298. doi: 10.3389/fmed.2022.1026298 (PMC9849690; doi:10.3389/fmed.2022.1026298)
Supplement: Supplementary file 4 [file Table_4.docx]

**Table S4:** Comparison of blood monocyte subsets in diabetic patients with/without obesity in sepsis

| **Parameter^⁜^** | **Diabetic** | | | | | |
| --- | --- | --- | --- | --- | --- | --- |
|  | **Non-obese** | | ***p*-value** | **Obese** | | ***p*-value** |
|  | **Non-sepsis**  **(n=23)** | **Septic**  **(n=13)** |  | **Non-sepsis**  **(n=21)** | **Septic**  **(n=24)** |  |
| **Age (years)** | 72 (63, 80) | 70 (61, 78) | 0.608 | 62 (52, 73) **†** | 68 (56, 76) | 0.290 |
| **Male/Female, n** | 19/4 | 13/0 | 0.559 | 11/10 **†** | 13/11**§§** | 0.710 |
| **BMI (kg/m^2^)** | 26 (24, 28) | 26 (25, 29) | 0.467 | 34 (31, 39) **†††****†** | 36 (32, 42) **§§§§** | 0.317 |
| **Monocytes%^a^** | 4.8 (3.4, 6.4) | 5.1 (3.1, 6.6) | 0.955 | 4.9 (2.8, 6.2) | 4.0 (2.6, 6.1) | 0.757 |
| **A:CD14^+^CD16^+^%^b^** | 32 (9.5, 47) | 47 (23, 59) | 0.312 | 39 (12, 71) | 24 (12, 55) | 0.919 |
| **B:CD14^+^CD16^−^%^b^** | 59 (41, 87) | 35 (21, 46) | **0.007** | 49 (19, 83) | 47 (31, 65) | 0.909 |
| **C:CD14^−^CD16^+^%^b^** | 1.3 (0.65, 3.1) | 1.9 (0.66, 2.5) | \|  \| \| --- \|   0.764 | 2.2 (1.1, 4.8) | 4.2 (1.6, 7.2) **§** | \|  \| \| --- \|   0.207 |
| **CD14 MFI-A** | 519 (362, 673) | 515 (146, 743) | 0.758 | 636 (424, 812) | 470 (295, 657) | 0.200 |
| **CD14 MFI-B** | 398 (287, 597) | 346 (165, 429) | 0.250 | 389 (272, 681) | 351 (242, 520) | 0.347 |
| **CD14 MFI-C** | 10 (9.4, 12) | 11 (8.7, 13) | 0.877 | 10 (8.1, 14) | 9.6 (7.7, 15) | 0.916 |
| **CD16 MFI-A** | 84 (44, 177) | 66 (42, 134) | 0.573 | 93 (40, 184) | 148 (45, 400) | 0.319 |
| **CD16 MFI-B** | 9.8 (6.1, 13) | 10 (6.4, 14) | 0.604 | 11 (5.9, 12) | 8.2 (5.6, 14) | 0.785 |
| **CD16 MFI-C** | 197 (70, 396) | 69 (31, 419) | 0.683 | 145 (72, 457) | 203 (103, 611) | 0.408 |
| **CD14^+^%^b^** | 93 (89, 96) | 83 (76, 92) | **0.012** | 93 (87, 96) | 85 (74, 92) | **0.0006** |
| **CD14 MFI** | 498 (401, 689) | 441 (170, 616) | 0.282 | 611 (411, 833) | 452 (387, 715) | 0.279 |
| **CD16^+^%^b^** | 35 (11, 51) | 48 (26, 61) | 0.379 | 39 (15, 72) | 32 (23, 50) | 0.813 |
| **CD16 MFI** | 93 (54, 212) | 67 (50, 139) | 0.478 | 105 (46, 202) | 274 (91, 428) **§** | **0.036** |
| **CD33^+^%^b^** | 97 (92, 98) | 97 (92, 99) | 0.861 | 99 (96, 99) | 96 (89, 97) | **0.002** |
| **CD33 MFI** | 538 (401, 792) | 473 (393, 858) | 0.753 | 604 (518, 801) | 562 (366, 705) | 0.165 |
| **HLA-DR^+^%^b^** | 82 (30, 90) | 63 (24, 75) | 0.272 | 73 (43, 84) | 58 (42, 71) | 0.310 |
| **HLA-DR MFI** | 213 (97, 466) | 234 (80, 364) | 0.778 | 140 (61, 288) | 314 (118, 340) | 0.238 |
| **Ratio A%/B%** | 0.5 (0.11, 1.3) | 1.6 (0.61, 2.3) | \|  \| \| --- \|   0.068 | 0.8 (0.15, 3.0) | 0.5 (0.24, 1.5) | \|  \| \| --- \|   0.987 |
| **CD163^+^%^b^** | 25 (4.7, 48) | 31 (19, 52) | 0.345 | 17 (1.8, 43) | 21 (6.0, 56) | 0.360 |
| **CD163 MFI** | 32 (25, 47) | 35 (30, 49) | 0.471 | 31 (24, 46) | 64 (32, 91) **§** | **0.005** |
| **CD206^+^%^b^** | 5.3 (2.0, 20) | 19 (9.8, 29) | 0.099 | 6.7 (1.9, 17) | 4.0 (2.3, 14) | 0.966 |
| **CD206 MFI** | 33 (24, 152) | 34 (25, 50) | 0.745 | 30 (21, 54) | 66 (32, 229) **§** | **0.035** |
| **Arg-1^+^%^b^** | 3.4 (1.8, 9.8) | 3.8 (2.1, 8.5) | 0.832 | 5.2 (3.6, 9.0) | 2.4 (1.1, 5.7) | 0.072 |
| **Arg-1 MFI** | 32 (24, 49) | 33 (26, 43) | 0.919 | 36 (22, 55) | 45 (31, 82) | 0.210 |

⁜ % a: monocytes among all leukocytes, b: monocyte subset in monocytes; n: number; BMI: body mass index; MFI: mean fluorescence intensity.

Monocyte subsets: CD14^+^CD16^+^ monocytes (A), CD14^+^CD16^−^ monocytes (B), CD14^−^CD16^+^ monocytes (C).

Results are medians (25th percentile, 75th percentile).

Given the sample size, the non-obese and obese diabetic groups were divided into non-septic and septic subgroups (combined sepsis and septic shock patients).

†*p* < 0.05, ††††*p* < 0.0001, vs. non-sepsis subgroup of diabetic and non-obese patients;

§*p* < 0.05, §§§§*p* < 0.0001, vs. septic subgroup of diabetic and non-obese patients.
