# Supplementary material for: Monocyte subtype expression patterns in septic patients with diabetes are distinct from patterns observed in obese patients
Source: Front Med (Lausanne). 2023 Jan 5;9:1026298. doi: 10.3389/fmed.2022.1026298 (PMC9849690; doi:10.3389/fmed.2022.1026298)
Supplement: Supplementary file 5 [file Table_5.docx]

**TableS5: Antibodies**

| **Specificity** | **Catalogno.** | **Format** | **Clone** | **Reactivity** | **Source** |
| --- | --- | --- | --- | --- | --- |
| lgG1 | 857.071.010 | FITC | B-Z1 | mouse | Diaclone |
| lgG2a | 857.082.010 | PE | B-Z2 | mouse | Diaclone |
| CD45/CD14 | 342408 | FITC/PE | 2D1/MΦP9 | human | BDBiosciences |
| CD14 | 954.771.010 | FITC | 8G3 | human | Diaclone |
| CD16 | 561313 | PE | B73.1 | human | BDBiosciences |
| CD33 | 366608 | PE | P67.6 | human | BioLegend |
| CD163 | 563697 | FITC | GHI/61 | human | BDBiosciences |
| CD206 | 130-095-220 | PE | DCN228 | human | MiltenyiBiotec |
| Arginase-1 | IC5868F | FITC | Polyclonal Sheep IgG | human/mouse | R&D Systems |
| IgG1 | 340442 | APC | X40 | human | BDBiosciences |
| HLA-DR | 307609 | APC | L-243 | human | BioLegend |
