# Supplementary material for: Monocyte subtype expression patterns in septic patients with diabetes are distinct from patterns observed in obese patients
Source: Front Med (Lausanne). 2023 Jan 5;9:1026298. doi: 10.3389/fmed.2022.1026298 (PMC9849690; doi:10.3389/fmed.2022.1026298)
Supplement: Supplementary file 6 [file Table_6.docx]

**Table S6. Cohen’s d analysis for non-diabetic/diabetic non-obese/obese specimens with or without sepsis.**

| **Cell population** | **Non-diabetic (NS Vs S)** | | | | | | | | | |  |
| --- | --- | --- | --- | --- | --- | --- | --- | --- | --- | --- | --- |
|  | **NS** | | | **S** | | | **NS Vs S** | | | |  |
|  | **Mean** | **SD** | **N** | **Mean** | **SD** | **N** | **Cohen’s *d*** | **CLES** | **95% CI** | ***p* - value** |  |
| Monocytes % | 4.6 | 2.4 | 116 | 4.2 | 2.1 | 27 | -0.17 | 0.548 | -0.59 - 0.249 | > 0.05 |  |
| CD14^+^ % | 88 | 14 | 116 | 78 | 17 | 27 | -0.685 | 0.686 | -1.111 - -0.259 | **< 0.05** |  |
| CD33^+^ % | 94 | 10 | 109 | 88 | 16 | 23 | -0.534 | 0.647 | -0.988 - -0.079 | > 0.05 |  |
| CD33 MFI | 630 | 374 | 109 | 472 | 316 | 23 | -0.433 | 0.62 | -0.886 - 0.02 | > 0.05 |  |
| CD163 MFI | 47 | 31 | 111 | 64 | 49 | 25 | 0.487 | 0.635 | 0.049 - 0.925 | **< 0.05** |  |
| CD14^+^CD16^−^ % | 53 | 28 | 116 | 43 | 25 | 27 | -0.364 | 0.602 | -0.785 - 0.057 | > 0.05 |  |
| CD14^+^CD16^+^ % | 35 | 28 | 116 | 36 | 29 | 27 | 0.035 | 0.51 | -0.383 - 0.454 | > 0.05 |  |
| CD14^−^CD16^+^ % | 2.4 | 3.2 | 116 | 2.3 | 2.8 | 27 | -0.032 | 0.509 | -0.451 - 0.387 | > 0.05 |  |
| CD14 MFI-B | 437 | 205 | 106 | 353 | 257 | 25 | -0.39 | 0.609 | -0.828 - 0.049 | **< 0.05** |  |
| CD16 MFI | 158 | 171 | 106 | 302 | 392 | 25 | 0.629 | 0.672 | 0.187 – 1.072 | > 0.05 |  |
| **Cell population** | **Non-diabetic (NS Vs SC)** | | | | | | | | | |  |
|  | **NS** | | | **SC** | | | **NS Vs SC** | | | |  |
|  | **Mean** | **SD** | **N** | **Mean** | **SD** | **N** | **Cohen’s *d*** | **CLES** | **95% CI** | ***p* - value** |  |
| Monocytes % | 4.6 | 2.4 | 116 | 2.2 | 1.2 | 11 | -1.031 | 0.767 | -1.663 - -0.4 | **< 0.05** |  |
| CD14^+^ % | 88 | 14 | 116 | 82 | 8.5 | 11 | -0.44 | 0.622 | -1.061 – 0.181 | **< 0.05** |  |
| CD33^+^ % | 94 | 10 | 109 | 89 | 3.9 | 10 | -0.517 | 0.643 | -1.168 - 0.134 | **< 0.05** |  |
| CD33 MFI | 630 | 374 | 109 | 447 | 387 | 10 | -0.488 | 0.635 | -1.139 - 0.163 | > 0.05 |  |
| CD163 MFI | 47 | 31 | 111 | 53 | 39 | 11 | 0.189 | 0.553 | -0.431 - 0.809 | > 0.05 |  |
| CD14^+^CD16^−^ % | 53 | 28 | 116 | 53 | 19 | 11 | 0 | 0.5 | -0.618 - 0.618 | > 0.05 |  |
| CD14^+^CD16^+^ % | 35 | 28 | 116 | 29 | 15 | 11 | -0.221 | 0.562 | -0.84 - 0.398 | > 0.05 |  |
| CD14^−^CD16^+^ % | 2.4 | 3.2 | 116 | 2.7 | 2.7 | 11 | 0.095 | 0.527 | -0.524 - 0.713 | > 0.05 |  |
| CD14 MFI-B | 437 | 205 | 106 | 379 | 172 | 10 | -0.286 | 0.58 | -0.936 – 0.363 | > 0.05 |  |
| CD16 MFI | 158 | 171 | 106 | 179 | 119 | 10 | 0.125 | 0.535 | -0.523 – 0.774 | > 0.05 |  |
| **Cell population** | **Non-diabetic (S Vs SC)** | | | | | | | | | |  |
|  | **S** | | | **SC** | | | **S Vs SC** | | | |  |
|  | **Mean** | **SD** | **N** | **Mean** | **SD** | **N** | **Cohen’s *d*** | **CLES** | **95% CI** | ***p* - value** |  |
| Monocytes % | 4.2 | 2.1 | 27 | 2.2 | 1.2 | 11 | -1.056 | 0.772 | -1.797 - -0.316 | **< 0.05** |  |
| CD14^+^ % | 78 | 17 | 27 | 82 | 8.5 | 11 | 0.264 | 0.574 | -0.439 - 0.968 | > 0.05 |  |
| CD33^+^ % | 88 | 16 | 23 | 89 | 3.9 | 10 | 0.073 | 0.521 | -0.669 - 0.816 | > 0.05 |  |
| CD33 MFI | 472 | 316 | 23 | 447 | 387 | 10 | -0.074 | 0.521 | -0.817 - 0.669 | > 0.05 |  |
| CD163 MFI | 64 | 49 | 25 | 53 | 39 | 11 | -0.238 | 0.567 | -0.949 - 0.474 | > 0.05 |  |
| CD14^+^CD16^−^ % | 43 | 25 | 27 | 53 | 19 | 11 | 0.426 | 0.618 | -0.282 - 1.133 | > 0.05 |  |
| CD14^+^CD16^+^ % | 36 | 29 | 27 | 29 | 15 | 11 | -0.27 | 0.576 | -0.974 - 0.433 | > 0.05 |  |
| CD14^−^CD16^+^ % | 2.3 | 2.8 | 27 | 2.7 | 2.7 | 11 | 0.144 | 0.541 | -0.558 - 0.846 | > 0.05 |  |
| CD14 MFI-B | 353 | 257 | 25 | 379 | 172 | 10 | 0.11 | 0.531 | -0.624 – 0.844 | > 0.05 |  |
| CD16 MFI | 302 | 392 | 25 | 179 | 119 | 10 | -0.362 | 0.601 | -1.1 – 0.377 | > 0.05 |  |
| **Cell population** | **Diabetic (NS Vs S)** | | | | | | | | | |  |
|  | **NS** | | | **S** | | | **NS Vs S** | | | |  |
|  | **Mean** | **SD** | **N** | **Mean** | **SD** | **N** | **Cohen’s *d*** | **CLES** | **95% CI** | ***p* - value** |  |
| Monocytes % | 4.7 | 2.0 | 44 | 6.1 | 2.6 | 22 | 0.632 | 0.673 | 0.109 - 1.155 | > 0.05 |  |
| CD14^+^ % | 91 | 6.4 | 44 | 82 | 20 | 22 | -0.714 | 0.693 | -1.24 - -0.188 | **< 0.05** |  |
| CD33^+^ % | 95 | 9.7 | 38 | 95 | 10 | 21 | 0 | 0.5 | -0.533 - 0.533 | > 0.05 |  |
| CD33 MFI | 587 | 252 | 38 | 611 | 256 | 21 | 0.095 | 0.527 | -0.439 - 0.628 | > 0.05 |  |
| CD163 MFI | 37 | 23 | 40 | 46 | 27 | 21 | 0.368 | 0.603 | -0.164 - 0.901 | > 0.05 |  |
| CD14^+^CD16^−^ % | 54 | 29 | 44 | 43 | 22 | 22 | -0.409 | 0.614 | -0.925 - 0.108 | > 0.05 |  |
| CD14^+^CD16^+^ % | 36 | 28 | 44 | 39 | 26 | 22 | 0.11 | 0.531 | -0.402 - 0.622 | > 0.05 |  |
| CD14^−^CD16^+^ % | 2.8 | 2.8 | 44 | 4.7 | 7.0 | 22 | 0.411 | 0.614 | -0.105 - 0.928 | > 0.05 |  |
| CD14 MFI-B | 463 | 290 | 37 | 336 | 189 | 20 | -0.489 | 0.635 | -1.041 - 0.062 | > 0.05 |  |
| CD16 MFI | 145 | 157 | 37 | 161 | 193 | 20 | 0.094 | 0.526 | -0.45 - 0.638 | > 0.05 |  |
| **Cell population** | **Diabetic (NS Vs SC)** | | | | | | | | | |  |
|  | **NS** | | | **SC** | | | **NS Vs SC** | | | |  |
|  | **Mean** | **SD** | **N** | **Mean** | **SD** | **N** | **Cohen’s *d*** | **CLES** | **95% CI** | ***p* - value** |  |
| Monocytes % | 4.7 | 2.0 | 44 | 2.9 | 1.2 | 15 | -0.98 | 0.756 | -1.592 - -0.368 | **< 0.05** |  |
| CD14^+^ % | 91 | 6.4 | 44 | 75 | 21 | 15 | -1.356 | 0.831 | -1.991 - -0.721 | **< 0.05** |  |
| CD33^+^ % | 95 | 9.7 | 38 | 85 | 20 | 15 | -0.749 | 0.702 | -1.364 - -0.135 | **< 0.05** |  |
| CD33 MFI | 587 | 252 | 38 | 445 | 270 | 15 | -0.552 | 0.652 | -1.159 - 0.054 | > 0.05 |  |
| CD163 MFI | 37 | 23 | 40 | 85 | 63 | 15 | 1.266 | 0.815 | 0.627 - 1.905 | **< 0.05** |  |
| CD14^+^CD16^−^ % | 54 | 29 | 44 | 42 | 23 | 15 | -0.434 | 0.621 | -1.025 - 0.157 | > 0.05 |  |
| CD14^+^CD16^+^ % | 36 | 28 | 44 | 32 | 25 | 15 | -0.147 | 0.541 | -0.733 - 0.44 | > 0.05 |  |
| CD14^−^CD16^+^ % | 2.8 | 2.8 | 44 | 3.5 | 3.7 | 15 | 0.23 | 0.565 | -0.358 - 0.817 | > 0.05 |  |
| CD14 MFI-B | 463 | 290 | 37 | 379 | 223 | 15 | -0.308 | 0.586 | -0.911 - 0.295 | > 0.05 |  |
| CD16 MFI | 145 | 157 | 37 | 398 | 480 | 15 | 0.882 | 0.734 | 0.259 - 1.506 | **< 0.05** |  |
| **Cell population** | **Diabetic (S Vs SC)** | | | | | | | | | |  |
|  | **S** | | | **SC** | | | **S Vs SC** | | | |  |
|  | **Mean** | **SD** | **N** | **Mean** | **SD** | **N** | **Cohen’s *d*** | **CLES** | **95% CI** | ***p* - value** |  |
| Monocytes % | 6.1 | 2.6 | 22 | 2.9 | 1.2 | 15 | -1.487 | 0.853 | -2.225 - -0.748 | **< 0.05** |  |
| CD14^+^ % | 82 | 20 | 22 | 75 | 21 | 15 | -0.343 | 0.596 | -1.004 - 0.318 | > 0.05 |  |
| CD33^+^ % | 95 | 10 | 21 | 85 | 20 | 15 | -0.669 | 0.682 | -1.349 - 0.012 | **< 0.05** |  |
| CD33 MFI | 611 | 256 | 21 | 445 | 270 | 15 | -0.634 | 0.673 | -1.313 - 0.045 | > 0.05 |  |
| CD163 MFI | 46 | 27 | 21 | 85 | 63 | 15 | 0.859 | 0.728 | 0.167 - 1.55 | > 0.05 |  |
| CD14^+^CD16^−^ % | 43 | 22 | 22 | 42 | 23 | 15 | -0.045 | 0.513 | -0.701 - 0.612 | > 0.05 |  |
| CD14^+^CD16^+^ % | 39 | 26 | 22 | 32 | 25 | 15 | -0.273 | 0.577 | -0.933 - 0.386 | > 0.05 |  |
| CD14^−^CD16^+^ % | 4.7 | 7.0 | 22 | 3.5 | 3.7 | 15 | -0.203 | 0.557 | -0.861 - 0.455 | > 0.05 |  |
| CD14 MFI-B | 336 | 189 | 20 | 379 | 223 | 15 | 0.211 | 0.559 | -0.461 - 0.882 | > 0.05 |  |
| CD16 MFI | 161 | 193 | 20 | 398 | 480 | 15 | 0.686 | 0.686 | -0.002 - 1.375 | **< 0.05** |  |
| **Cell population** | **Non-diabetic and non-obese (NS Vs S)** | | | | | | | | | |  |
|  | **NS** | | | **S** | | | **NS Vs S** | | | |  |
|  | **Mean** | **SD** | **N** | **Mean** | **SD** | **N** | **Cohen’s *d*** | **CLES** | **95% CI** | ***p* - value** |  |
| Monocytes % | 4.8 | 2.5 | 81 | 3.9 | 1.6 | 16 | -0.378 | 0.605 | -0.917 - 0.161 | > 0.05 |  |
| CD14^+^ % | 87 | 16 | 81 | 76 | 20 | 16 | -0.659 | 0.679 | -1.203 - -0.115 | **< 0.05** |  |
| CD33^+^ % | 94 | 12 | 76 | 83 | 19 | 14 | -0.829 | 0.721 | -1.412 - -0.246 | > 0.05 |  |
| CD33 MFI | 680 | 401 | 76 | 470 | 319 | 14 | -0.538 | 0.648 | -1.114 - 0.037 | > 0.05 |  |
| CD163 MFI | 47 | 33 | 80 | 66 | 56 | 16 | 0.505 | 0.639 | -0.037 - 1.046 | > 0.05 |  |
| CD14^+^CD16^−^ % | 54 | 29 | 81 | 39 | 21 | 16 | -0.538 | 0.648 | -1.079 - 0.004 | > 0.05 |  |
| CD14^+^CD16^+^ % | 33 | 29 | 81 | 38 | 25 | 16 | 0.176 | 0.55 | -0.361 - 0.713 | > 0.05 |  |
| CD14^−^CD16^+^ % | 2.3 | 3.3 | 81 | 2.3 | 3.1 | 16 | 0 | 0.5 | -0.536 - 0.536 | > 0.05 |  |
| CD14 MFI-B | 447 | 221 | 74 | 297 | 196 | 16 | -0.691 | 0.688 | -1.241 - -0.142 | **< 0.05** |  |
| CD16 MFI | 163 | 186 | 74 | 348 | 458 | 16 | 0.729 | 0.697 | 0.178 - 1.279 | > 0.05 |  |
| **Cell population** | **Non-diabetic and non-obese (NS Vs SC)** | | | | | | | | | |  |
|  | **NS** | | | **SC** | | | **NS Vs SC** | | | |  |
|  | **Mean** | **SD** | **N** | **Mean** | **SD** | **N** | **Cohen’s *d*** | **CLES** | **95% CI** | ***p* - value** |  |
| Monocytes % | 4.8 | 2.5 | 81 | 2.3 | 1.2 | 10 | -1.041 | 0.769 | -1.715 - -0.367 | **< 0.05** |  |
| CD14^+^ % | 87 | 16 | 81 | 83 | 8.4 | 10 | -0.26 | 0.573 | -0.918 - -0.398 | > 0.05 |  |
| CD33^+^ % | 94 | 12 | 76 | 89 | 4.2 | 9 | -0.435 | 0.621 | -1.13 - -0.259 | **< 0.05** |  |
| CD33 MFI | 680 | 401 | 76 | 441 | 410 | 9 | -0.552 | 0.652 | -1.159 - 0.054 | > 0.05 |  |
| CD163 MFI | 47 | 33 | 80 | 58 | 36 | 10 | 0.33 | 0.592 | -0.329 - 0.989 | > 0.05 |  |
| CD14^+^CD16^−^ % | 54 | 29 | 81 | 54 | 20 | 10 | 0 | 0.5 | -0.657 - 0.657 | > 0.05 |  |
| CD14^+^CD16^+^ % | 33 | 29 | 81 | 30 | 15 | 10 | -0.108 | 0.53 | -0.765 - 0.55 | > 0.05 |  |
| CD14^−^CD16^+^ % | 2.3 | 3.3 | 81 | 2.9 | 2.7 | 10 | 0.185 | 0.552 | -0.473 - 0.842 | > 0.05 |  |
| CD14 MFI-B | 447 | 221 | 74 | 380 | 182 | 9 | -0.308 | 0.586 | -1.002 - 0.385 | > 0.05 |  |
| CD16 MFI | 163 | 186 | 74 | 174 | 125 | 9 | 0.061 | 0.517 | -0.631 - 0.753 | > 0.05 |  |
| **Cell population** | **Non-diabetic and non-obese (S Vs SC)** | | | | | | | | | |  |
|  | **S** | | | **SC** | | | **S Vs SC** | | | |  |
|  | **Mean** | **SD** | **N** | **Mean** | **SD** | **N** | **Cohen’s *d*** | **CLES** | **95% CI** | ***p* - value** |  |
| Monocytes % | 3.9 | 1.6 | 16 | 2.3 | 1.2 | 10 | -1.094 | 0.78 | -1.938 - -0.25 | > 0.05 |  |
| CD14^+^ % | 76 | 20 | 16 | 83 | 8.4 | 10 | 0.421 | 0.617 | -0.377 - 1.219 | > 0.05 |  |
| CD33^+^ % | 83 | 19 | 14 | 89 | 4.2 | 9 | 0.395 | 0.61 | -0.45 - 1.241 | > 0.05 |  |
| CD33 MFI | 470 | 319 | 14 | 441 | 410 | 9 | -0.595 | 0.663 | -1.291 - 0.102 | > 0.05 |  |
| CD163 MFI | 66 | 56 | 16 | 58 | 36 | 10 | -0.162 | 0.546 | -0.953 - 0.63 | > 0.05 |  |
| CD14^+^CD16^−^ % | 39 | 21 | 16 | 54 | 20 | 10 | 0.727 | 0.696 | -0.087 - 1.542 | > 0.05 |  |
| CD14^+^CD16^+^ % | 38 | 25 | 16 | 30 | 15 | 10 | -0.367 | 0.602 | -1.163 - 0.429 | > 0.05 |  |
| CD14^−^CD16^+^ % | 2.3 | 3.1 | 16 | 2.9 | 2.7 | 10 | 0.203 | 0.557 | -0.589 - 0.995 | > 0.05 |  |
| CD14 MFI-B | 297 | 196 | 16 | 380 | 182 | 9 | 0.434 | 0.621 | -0.391 - 1.259 | > 0.05 |  |
| CD16 MFI | 348 | 458 | 16 | 174 | 125 | 9 | -0.461 | 0.628 | -1.288 - 0.365 | > 0.05 |  |
| **Cell population** | **Non-diabetic and non-obese (NS Vs SS)** | | | | | | | | | |  |
|  | **NS** | | | **SS** | | | **NS Vs SS** | | | |  |
|  | **Mean** | **SD** | **N** | **Mean** | **SD** | **N** | **Cohen’s *d*** | **CLES** | **95% CI** | ***p* - value** |  |
| Monocytes % | 4.8 | 2.5 | 81 | 3.3 | 1.6 | 26 | -0.647 | 0.676 | -1.097 - -0.197 | **< 0.05** |  |
| CD14^+^ % | 87 | 16 | 81 | 79 | 16 | 26 | -0.5 | 0.638 | -0.947 - -0.053 | **< 0.05** |  |
| CD33^+^ % | 94 | 12 | 76 | 85 | 15 | 23 | -0.706 | 0.691 | -1.183 - -0.23 | **< 0.05** |  |
| CD33 MFI | 680 | 401 | 76 | 459 | 349 | 23 | -0.567 | 0.656 | -1.04 - -0.094 | **< 0.05** |  |
| CD163 MFI | 47 | 33 | 80 | 63 | 49 | 26 | 0.427 | 0.619 | -0.019 - -0.873 | > 0.05 |  |
| CD14^+^CD16^−^ % | 54 | 29 | 81 | 45 | 21 | 26 | -0.33 | 0.592 | 0.774 - 0.114 | > 0.05 |  |
| CD14^+^CD16^+^ % | 33 | 29 | 81 | 35 | 22 | 26 | 0.073 | 0.521 | -0.369 - 0.515 | > 0.05 |  |
| CD14^−^CD16^+^ % | 2.3 | 3.3 | 81 | 2.6 | 2.9 | 26 | 0.093 | 0.526 | -0.348 - 0.535 | > 0.05 |  |
| CD14 MFI-B | 447 | 221 | 74 | 327 | 191 | 25 | -0.561 | 0.654 | -1.021 - -0.101 | **< 0.05** |  |
| CD16 MFI | 163 | 186 | 74 | 286 | 379 | 25 | 0.496 | 0.637 | 0.037 - 0.954 | > 0.05 |  |
| **Cell population** | **Non-diabetic and obese (NS Vs SS)** | | | | | | | | | |  |
|  | **NS** | | | **SS** | | | **NS Vs SS** | | | |  |
|  | **Mean** | **SD** | **N** | **Mean** | **SD** | **N** | **Cohen’s *d*** | **CLES** | **95% CI** | ***p* - value** |  |
| Monocytes % | 4.3 | 2.0 | 35 | 4.3 | 2.7 | 12 | 0 | 0.5 | -0.656 - 0.656 | > 0.05 |  |
| CD14^+^ % | 89 | 7.6 | 35 | 81 | 12 | 12 | -0.901 | 0.738 | -1.581 - -0.22 | **< 0.05** |  |
| CD33^+^ % | 96 | 4.3 | 33 | 94 | 4.6 | 10 | -0.458 | 0.627 | -1.172 - 0.256 | > 0.05 |  |
| CD33 MFI | 516 | 273 | 33 | 477 | 313 | 10 | -0.138 | 0.539 | -0.846 - 0.57 | > 0.05 |  |
| CD163 MFI | 46 | 24 | 31 | 54 | 38 | 10 | 0.287 | 0.58 | -0.428 - 1.003 | > 0.05 |  |
| CD14^+^CD16^−^ % | 50 | 25 | 35 | 49 | 28 | 12 | -0.039 | 0.511 | -0.695 - 0.617 | > 0.05 |  |
| CD14^+^CD16^+^ % | 39 | 26 | 35 | 34 | 34 | 12 | -0.178 | 0.55 | -0.834 - 0.479 | > 0.05 |  |
| CD14^−^CD16^+^ % | 2.4 | 2.8 | 35 | 2.0 | 2.4 | 12 | -0.148 | 0.542 | -0.804 - 0.509 | > 0.05 |  |
| CD14 MFI-B | 413 | 161 | 32 | 445 | 312 | 10 | 0.156 | 0.544 | -0.555 - 0.867 | > 0.05 |  |
| CD16 MFI | 146 | 129 | 32 | 220 | 222 | 10 | 0.478 | 0.632 | -0.24 - 1.195 | > 0.05 |  |
| **Cell population** | **Diabetic and non-obese (NS Vs SS)** | | | | | | | | | |  |
|  | **NS** | | | **SS** | | | **NS Vs SS** | | | |  |
|  | **Mean** | **SD** | **N** | **Mean** | **SD** | **N** | **Cohen’s *d*** | **CLES** | **95% CI** | ***p* - value** |  |
| Monocytes % | 4.8 | 1.9 | 23 | 4.9 | 2.4 | 13 | 0.048 | 0.513 | -0.632 - -0.728 | > 0.05 |  |
| CD14^+^ % | 91 | 7.4 | 23 | 75 | 28 | 13 | -0.906 | 0.739 | -1.617 - -0.194 | **< 0.05** |  |
| CD33^+^ % | 93 | 13 | 21 | 87 | 25 | 13 | -0.325 | 0.591 | -1.021 - -0.371 | > 0.05 |  |
| CD33 MFI | 551 | 259 | 21 | 550 | 310 | 13 | -0.004 | 0.501 | -0.695 - 0.688 | > 0.05 |  |
| CD163 MFI | 41 | 26 | 21 | 38 | 11 | 12 | -0.137 | 0.539 | -0.847 - 0.573 | > 0.05 |  |
| CD14^+^CD16^−^ % | 59 | 26 | 23 | 34 | 18 | 13 | -1.064 | 0.774 | -1.787 - -0.341 | **< 0.05** |  |
| CD14^+^CD16^+^ % | 32 | 25 | 23 | 41 | 23 | 13 | 0.37 | 0.603 | -0.315 - 1.056 | > 0.05 |  |
| CD14^−^CD16^+^ % | 2.5 | 2.7 | 23 | 1.7 | 1.2 | 13 | -0.35 | 0.598 | -1.035 - 0.335 | > 0.05 |  |
| CD14 MFI-B | 427 | 245 | 20 | 309 | 153 | 13 | -0.551 | 0.652 | -1.262 - 0.16 | > 0.05 |  |
| CD16 MFI | 130 | 107 | 20 | 97 | 59 | 13 | -0.361 | 0.601 | -1.065 - 0.343 | > 0.05 |  |
| **Cell population** | **Diabetic and obese (NS Vs SS)** | | | | | | | | | |  |
|  | **NS** | | | **SS** | | | **NS Vs SS** | | | |  |
|  | **Mean** | **SD** | **N** | **Mean** | **SD** | **N** | **Cohen’s *d*** | **CLES** | **95% CI** | ***p* - value** |  |
| Monocytes % | 4.5 | 2.1 | 21 | 4.8 | 2.9 | 24 | 0.117 | 0.533 | -0.469 - -0.703 | > 0.05 |  |
| CD14^+^ % | 92 | 5.1 | 21 | 81 | 15 | 24 | -0.956 | 0.75 | -1.574 - -0.338 | **< 0.05** |  |
| CD33^+^ % | 98 | 2.0 | 17 | 93 | 6.6 | 23 | -0.964 | 0.752 | -1.626 - -0.302 | **< 0.05** |  |
| CD33 MFI | 632 | 243 | 17 | 537 | 253 | 23 | -0.382 | 0.606 | -1.014 - 0.251 | > 0.05 |  |
| CD163 MFI | 33 | 18 | 19 | 74 | 56 | 24 | 0.94 | 0.747 | 0.306 - 1.574 | **< 0.05** |  |
| CD14^+^CD16^−^ % | 48 | 33 | 21 | 47 | 23 | 24 | -0.036 | 0.51 | -0.621 - 0.55 | > 0.05 |  |
| CD14^+^CD16^+^ % | 40 | 30 | 21 | 34 | 26 | 24 | -0.215 | 0.56 | -0.802 ­- 0.373 | > 0.05 |  |
| CD14^−^CD16^+^ % | 3.2 | 2.9 | 21 | 5.6 | 6.9 | 24 | 0.443 | 0.623 | -0.15 - 1.036 | > 0.05 |  |
| CD14 MFI-B | 505 | 338 | 17 | 381 | 225 | 22 | -0.444 | 0.623 | -1.084 - 0.197 | > 0.05 |  |
| CD16 MFI | 163 | 203 | 17 | 361 | 425 | 22 | 0.571 | 0.657 | -0.075 – 1.216 | **< 0.05** |  |
| NS = Non-sepsis \| S = Sepsis \| SC = Septic shock \| SS = Septic (Sepsis and septic shock combined) \| SD = Standard deviation \| N = Sample size \| B = CD14^+^CD16^−^ monocytes \| CLES = Common language effect size \| CI = Confidence interval \| *p* values < 0.05 considered statistically significant | | | | | | | | | | | |
