# Supplementary figures and images for: Monocyte subtype expression patterns in septic patients with diabetes are distinct from patterns observed in obese patients
Source: Front Med (Lausanne). 2023 Jan 5;9:1026298. doi: 10.3389/fmed.2022.1026298 (PMC9849690; doi:10.3389/fmed.2022.1026298)

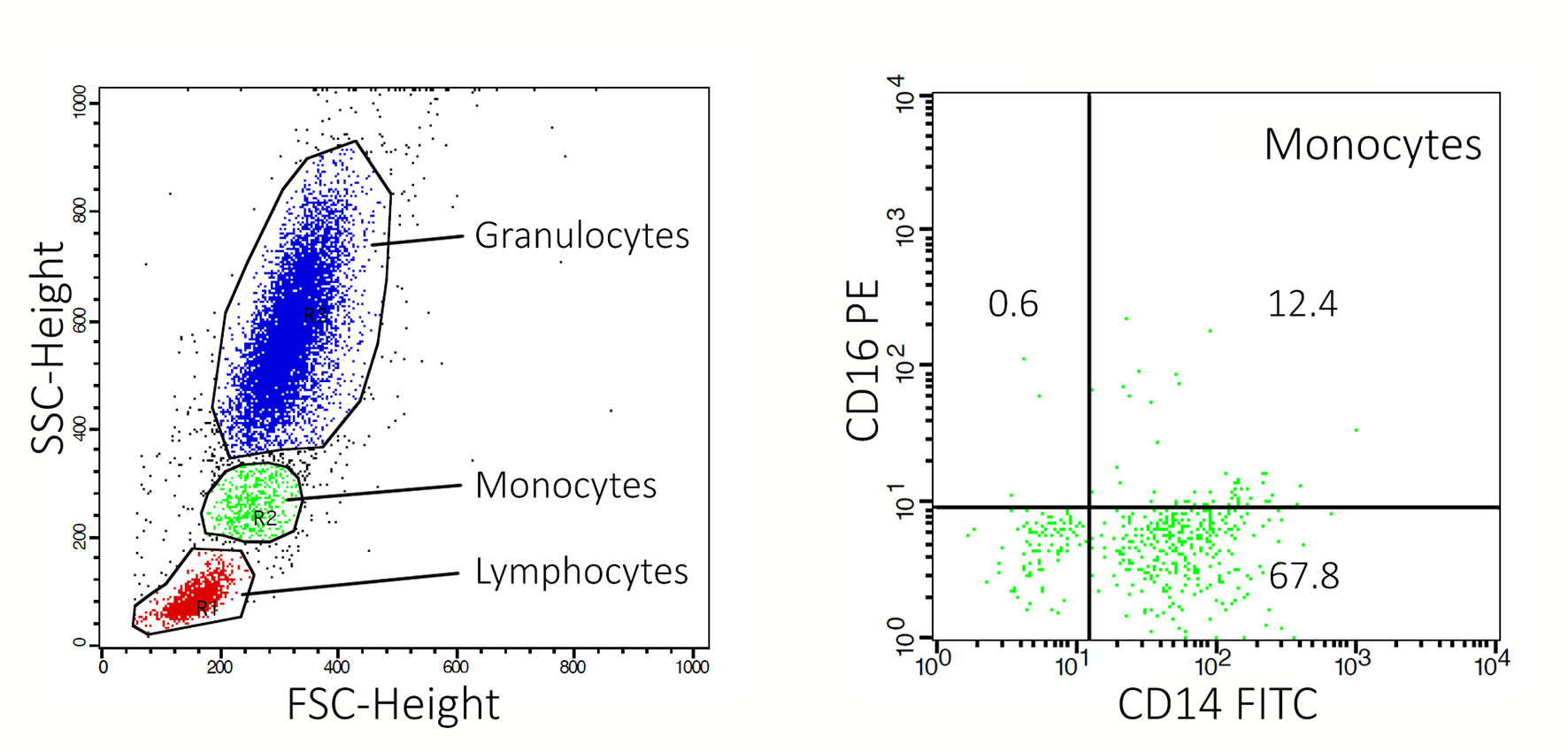

Supplement: Supplementary Figure 1 — CD14 and CD16 staining of peripheral blood monocytes in one patient specimen. (A) Identification of lymphocytes (Rl gate), monocytes (R2 gate), and granulocytes (R3 gate) displayed in a representative FSC/SSC dot plot. (B) Staining by FITC-conjugated anti-CD 14 and PE-conjugated anti-CD 16 antibodies. Monocytes were gated based on their light scatter profiles, and subsets identified by the fluorescence gates of CD14-FITC and CD16-PE: CD14–CD16– monocytes (lower right gate), CD14+CD16– monocytes (upper right gate), and CD14–CD16+ monocytes (upper left gate), with respective percentage in the box. [file Image_1.tif]
